# Medication-induced osteonecrosis of the jaw: a review of cases from the Food and Drug Administration Adverse Event Reporting System (FAERS)

**DOI:** 10.1186/s40360-023-00657-y

**Published:** 2023-03-06

**Authors:** Hardeep S. Ahdi, Thomas Adam Wichelmann, Sasirekha Pandravada, Eli D. Ehrenpreis

**Affiliations:** Department of Internal Medicine, Advocate Lutheran General Hospital, 1775 Dempster Street, Park Ridge, IL 60068 USA

**Keywords:** Osteonecrosis, Adverse drug reaction, Osteonecrosis, Bisphosphonates, Denosumab, m-TOR inhibitors, Antiangiogenics

## Abstract

**Background:**

Osteonecrosis of the jaw (ONJ) is a rare but serious adverse drug reaction (ADR) commonly associated with bisphosphonate and denosumab therapy. Prior research utilized an online, public FDA Adverse Event Reporting System (FAERS) Database to explore this ADR. This data identified and described several novel medications associated with ONJ. Our study aims to build upon the prior findings, reporting trends of medication induced ONJ over time and identifying newly described medications.

**Methods:**

We searched the FAERS database for all reported cases of medication related osteonecrosis of the jaw (MRONJ) from 2010 to 2021. Cases lacking patient age or gender were excluded. Only adults (18 +) and reports from Healthcare Professions were included. Duplicate cases were removed. The top 20 medications were identified and described for April 2010-December 2014 and April 2015-January 2021.

**Results:**

Nineteen thousand six hundred sixty-eight cases of ONJ were reported to the FAERS database from 2010–2021. 8,908 cases met inclusion criteria. 3,132 cases were from 2010–2014 and 5,776 cases from 2015–2021. Within the cases from 2010–2014, 64.7% were female and 35.3% were male, and the average age was 66.1 ± 11.1 years. Between 2015–2021, 64.3% were female and 35.7% were male, and the average age was 69.2 ± 11.5 years. Review of the 2010–2014 data identified several medications and drug classes associated with ONJ not previously described. They include lenalidomide, corticosteroids (prednisolone and dexamethasone), docetaxel and paclitaxel, letrozole, methotrexate, imatinib, and teriparatide. Novel drugs and classes described between 2015–2021 include palbociclib, pomalidomide, radium 223, nivolumab, and cabozantinib.

**Discussion:**

While stricter inclusion criteria and removal of duplicate cases led to fewer overall identified cases of MRONJ when compared to prior research, our data represents a more reliable analysis of MRONJ reports to the FAERS database. Denosumab was the most frequently reported medication associated with ONJ. While unable to imply incidence rates from our data due to the nature of the FAERS database, our findings provide further description of the various medications associated with ONJ and elucidate patient demographics associated with the ADR. Additionally, our study identifies cases of several newly described drugs and drug classes that have not been previously described in literature.

## Background

Medication-related osteonecrosis of the jaw (MRONJ) is a rare but serious adverse drug reaction (ADR) most frequently associated with bisphosphonate pharmaceuticals used in the treatment of osteoporosis, malignancy associated metabolic bone lesions, and Paget disease of bone [1, 2]. A Phase III trial of bisphosphonate therapy revealed oral adverse events in 4.8% of the total study population with 1.6% cases of positively adjudicated ONJ [2]. More recent data suggests a 0.7—6.7% risk of MRONJ amongst cancer patients exposed to bisphosphonate therapy, which was approximately 50–100 times higher than those treated with placebo [3]. The American Association of Oral and Maxillofacial Surgeons (AAOMS) recommends consideration of MRONJ in patients with current or previous treatment with antiresorptive or antiangiogenic agents, exposed bone or bone that is probable via an intraoral or extraoral fistula in the maxillofacial region that is persistent for over eight weeks, and having no history of prior jaw radiation therapy or metastatic involvement of the jaw [3].

The AAOMS highlights three classes of medications related to MRONJ: bisphosphonates, RANK ligand inhibitors, and antiangiogenic medications [3]. However, a recent study identified several additional medications and classes of medications associated with ONJ [4]. In 2015, that study explored MRONJ utilizing the United States Food and Drug Administration’s Adverse Event Reporting System (FAERS) database [4]. The FAERS database is a large database of voluntarily reported ADRs associated with post-marked, FDA-approved medications as well as natural substances, vaccines, and medical devices. This database has been utilized in many high quality studies and is the gold standard method for identifying “signals,” and previously undescribed ADRs [4]. While most cases were attributed to bisphosphonate or denosumab therapy, there were several additional and surprising medications that were identified. While an excellent framework for future research, the prior study had two limitations with the methodology that impacted the results. First, the study did not address the inherent limitation of duplicate cases within the FAERS database. As prior research has identified, the FAERS database has a significant degree of duplication amongst reported cases likely secondary to the non-uniformity of reporting, accuracy and application of data is significantly impacted by the data review process [5]. Unfortunately, this is a significant limitation to the data analysis approach performed in prior research. In addition, the former study utilized a narrow time frame of case reports (2010–2014), with analysis ending at the time of publication. Our study aims to update, elaborate, and build off the prior findings of the initial FAERS study by using strict data curation with removal of duplicate cases reports for the same time frame as well as inclusion of additional cases within an expanded time frame.

## Methods

**Fig. 1:**
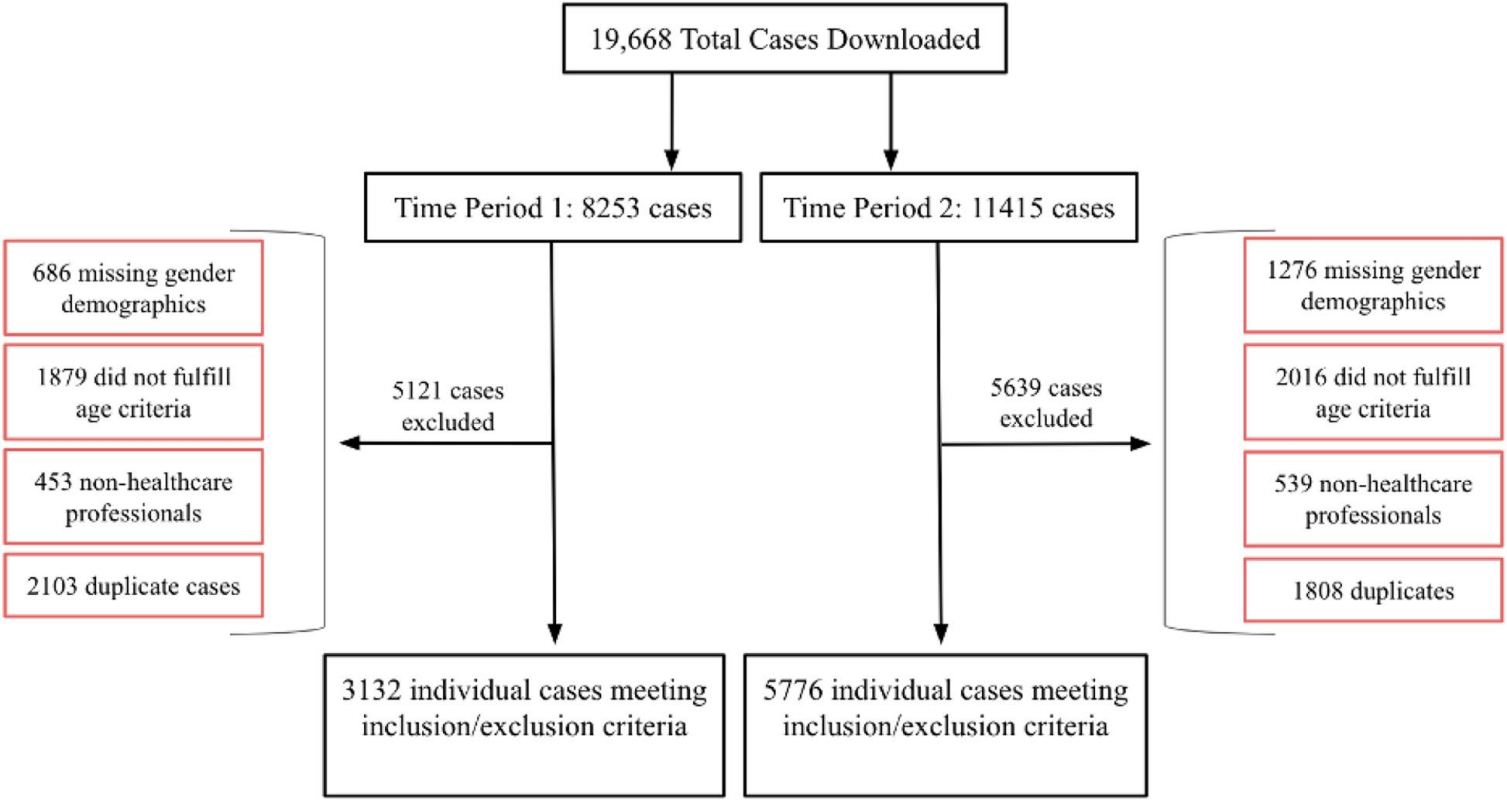
Illustrates a flow chart representation of our inclusion criteria when comparing time period 1 (2010–2014 data set) to time period 2 (2015–2021 data set)

By May 2021, the FAERS database contained a total of 22,002,078 reported cases of ADRs. Our methods include a wide search of the FAERS database for cases of medication induced ONJ with a total number of cases being reviewed at 19,668. The search strategy was that within the FAERS database we specified a “Search by Reaction Term” and looked up “Osteonecrosis of jaw.” We then selected a time frame with the first time period being similar to that of the original study from April 1, 2010 to December 31, 2014 (labeled as “time period 1”) and the second time period being from April 1, 2015 to Jan 12, 2021 (labeled as “time period 2”). Time periods 1 and 2 contained 8,253 and 11,415 cases respectively. We then determined our inclusion criteria for both sets of data. In doing so, we excluded patients that did not have gender listed as part of the patient demographics (ie, gender labeled as “Not specified”). Secondly, we did not include cases for patients below the age of 18, as we were primarily evaluating adult data. Thirdly, since the FAERS database allows consumers, healthcare professionals, and non-specified individuals to report their findings, we only included those reported by healthcare professionals allowing for increased accuracy of reporting. Lastly, we excluded duplicate cases using previously utilized methods, a multistep review process [4]. This involved identifying common patient characteristics from the FAERS database including patient age, gender, weight, event date, location (by country), drug reactions. After identifying patients with the same common characteristics, it was assumed these were duplicate patient information and thus, the duplicates were removed. This inclusion criteria nearly reduced the number of cases by nearly 60%, resulting in 3,132 cases in time period 1 and 5,776 in time period 2. This breakdown can be seen as a flow chart representation in Fig. 1. The top 20 medications were identified and described for these two different time periods. An analysis was performed on the collective patient demographics of medication-induced ONJ reports as well as for the two timeline ranges. Specific information obtained for this review included patient age, patient gender, associated medications, and indication for medication use. Descriptive statistics for all categorical [N (%)] variables were determined on all patients’ demographic and clinical characteristics. This project was submitted for review through Advocate Health and was waived as this study was deemed non-human subject related research.

## Results

**Fig. 2:**
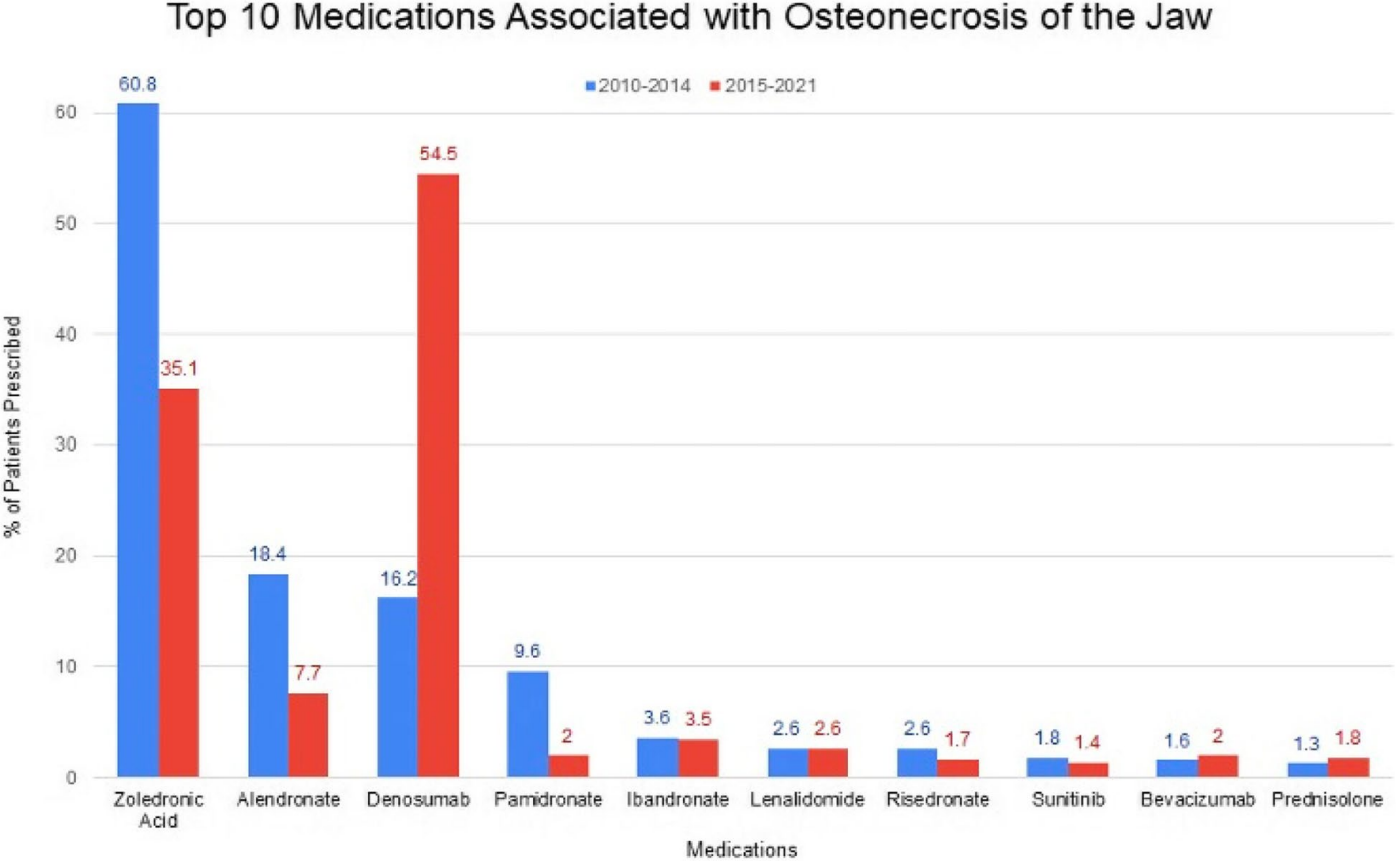
Illustrates a graphical representation of the percentage of cases for each of the ten most commonly reported medications associated with ONJ when comparing 2010–2014 data set to 2015-present. Blue represents the former 2010–2014 data set, whereas red represents the 2015–2021 data set

**Fig. 3:**
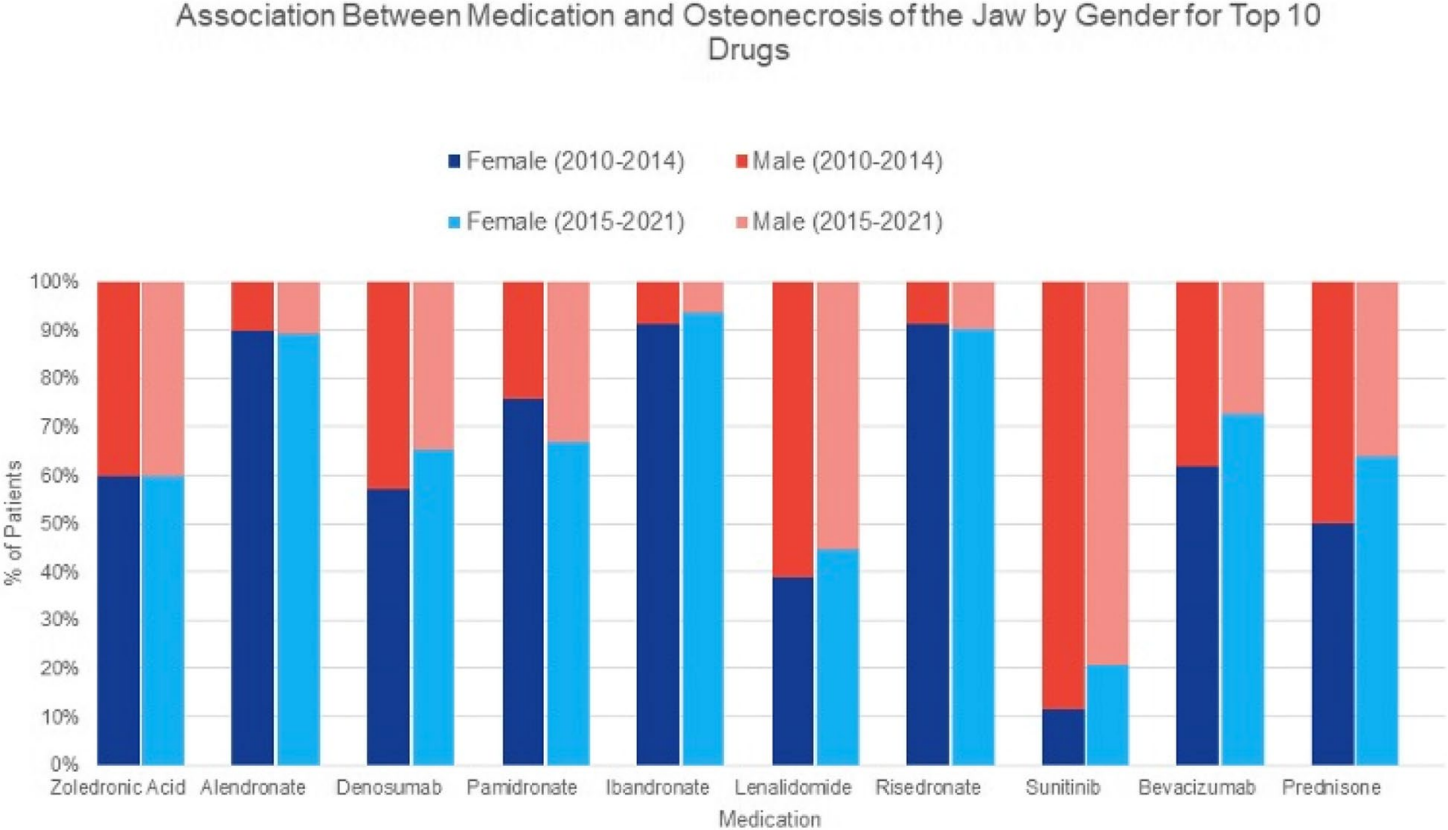
Illustrates a graphical representation of gender distribution when comparing 2010–2014 data set to 2015–2021

Nineteen thousand six hundred sixty-eight cases of ONJ were reported to the FAERS database from April 2010 to Jan 2021. Of these, 8908 cases met inclusion criteria. 3,132 cases were reported from 2010 to 2014 and 5,776 cases from 2015 to 2021. Average patient age from 2010 to 2014 was 66.1 ± 11.1 years, while average age from 2015 to present was 69.2 ± 11.5. Gender distribution during the 2010–2014 time frame included 2,026 females (64.7%) and 1,106 males (35.3%). Gender distribution during the 2015–2021 time frame included 3,712 females (64.3%) and 2,064 males (35.7%). We found a total of 432 cases (2.2% of total cases during our studied time interval) that listed mortality as the outcome on the database, 182 cases (5.8%) were during 2010–2014 and 250 cases (4.3%) was during 2015–2021. The twenty most reported medications with their associated drug class and indication from 2010–2014 and from 2015–2021 are shown in Table 1. As illustrated, the top 5 drugs in each time frame share mutual drugs including zoledronic acid, alendronic acid, denosumab, and ibandronate however they appear to differ in their rankings. Pamidronic acid, however, does not appear within the top five rankings during the more present time frame, with increasing cases of MRONJ from lenalidomide. The database confirms that bisphosphonates and RANKL-i comprised the majority of cases. Table 2 shows demographic information for each of the twenty most commonly reported medications during those time frames. The median age of zoledronic acid induced MRONJ in both time frames were very similar, (65 and 68, respectively). A similar percentage of females, (59.7 and 59.8, respectively) was also seen for both time range periods. A large proportion of females were noted with the use of denosumab, as it was noted to be the top drug associated with MRONJ during the latter time frame with as many as 65.3% compared to 57.3% in the former group. Figure 2 is a graphical representation illustrating the cases attributed to the ten most commonly reported drugs associated with ONJ during the two study period times. The figure demonstrates that each of the ten drugs listed in the 2010–2014 also appeared in the 2015–2021 time period, however in modified order. Figure 3 further illustrates the data by providing a graphical representation of gender distribution among the two different time periods. Table 3 illustrates a two-by-two contingency table that was utilized when calculating the reporting odds ratio (ROR) for the top ten drugs in each time period as seen in Tables 4 and 5. Review of the 2010–2014 data set identified several medications and drug classes associated with ONJ not previously described. They include lenalidomide, corticosteroids (prednisolone and dexamethasone), docetaxel and paclitaxel, letrozole, methotrexate, imatinib, and teriparatide. Novel drugs and classes described between 2015–2021 include palbociclib, pomalidomide, radium 223, nivolumab, and cabozantinib.

**Table 1:** This table depicts the number of cases of MRONJ reported to the FAERS database for each of the twenty most commonly reported medications from 2010 to 2014 and from 2015 to 2021

**2010 to 2014**				**2015 to 2021**			
**Medication (Drug Class):**	**Drug Indication:**	**# of ONJ Cases**	**% of total reported**	**Medication:**	**Drug Indication:**	**# of ONJ Cases**	**% of total reported**
1. Zoledronic Acid (BP)	Osteoporosis	1903	60.8	1. Denosumab (RANKL-i)	Osteoporosis	3148	54.5
2. Alendronate (BP)	Osteoporosis	576	18.4	2. Zoledronic Acid (BP)	Osteoporosis	2027	35.1
3. Denosumab (RANKL-i)	Osteoporosis	506	16.2	3. Alendronate (BP)	Osteoporosis	447	7.7
4. Pamidronate (BP)	Osteoporosis	301	9.6	4. Ibandronate (BP)	Osteoporosis	200	3.5
5. Ibandronate (BP)	Osteoporosis	112	3.6	5. Lenalidomide (immunomodulator w multiple MOA)	Cancer; immunomodulator with multiple MOA	150	2.6
6. Lenalidomide (immunomodulator w multiple MOA)	Cancer; immunomodulator with multiple MOA	82	2.6	6. Pamidronate (BP)	Osteoporosis	115	2.0
7. Risedronate (BP)	Osteoporosis	81	2.6	7. Bevacizumab (anti-VEGF)	Cancer; monoclonal anti-VEGF	114	2.0
8. Sunitinib (TKI, antiangiogenic)	Cancer; tyrosine kinase inhibitor	56	1.8	8. Prednisolone (CS)	Variety of different indications; many different MOA	103	1.8
9. Bevacizumab (anti-VEGF)	Cancer; monoclonal anti-VEGF	50	1.6	9. Risedronate (BP)	Osteoporosis	101	1.7
10. Prednisolone (CS)	Variety of different indications; many different MOA	42	1.3	10. Dexamethasone (CS)	Variety of different indications; many different MOA	99	1.7
11. Dexamethasone (CS)	Variety of different indications; many different MOA	35	11.1	11. Everolimus (mTOR i)	Cancer; immunomodulator	90	1.6
12. Docetaxel (MT depolymerizer)	Cancer; MT depolymerization	24	0.8	12. Sunitinib (TKI, antiangiogenesis)	Cancer; tyrosine kinase inhibitor	78	1.4
13. Letrozole (aromatase inhibitor)	Cancer; hormonal effects	15	0.5	13. Palbociclib (CDK 4/6 inhibitor)	Cancer; hormonal effects	74	1.3
14. Methotrexate (antimetabolite)	Cancer; cytotoxic agent	15	0.5	14. Docetaxel (MT depolymerizer)	Cancer; MT depolymerization	54	0.9
15. Everolimus (mTOR inhibitor)	Cancer; immunomodulator	12	0.4	15. Methotrexate (antimetabolite)	Cancer; cytotoxic agent	51	0.9
16. Paclitaxel (MT depolymerizer)	Cancer; MT depolymerization	9	0.3	16. Prednisone (CS)	Variety of different indications; many different MOA	47	0.8
17. Imatinib (TKI)	Cancer; tyrosine kinase inhibitor	8	0.3	17. Pomalidomide (unclear; multiple MOA	Cancer; immunomodulator	29	0.5
18. Sorafenib (TKI, anti VEGF)	Cancer; immunomodulator	8	0.3	18. Radium 223 (emits high energy particles inducing cytotoxic DNA ds breaks in cells)	Cancer; radiotherapy	25	0.4
19. Teriparatide (rPTH)	Osteoporosis; hormonal effects	7	0.2	19. Nivolumab (PD Rc-1 inhibitor)	Cancer; immunomodulator	23	0.4
20. Temsirolimus (mTOR inhibitor)	Cancer; immunomodulator	6	0.2	20. Cabozantinib (TKI)	Cancer; tyrosine kinase inhibitor	22	0.4

Key: *BP* Bisphosphonate, *RANKL-i* Receptor activator of nuclear factor kappa-B ligand inhibitor, *MOA* Mechanism of action, *TKI* Tyrosine kinase inhibitor, *VEGF* Vascular endothelial growth factor, *CS* Corticosteroid, *MT* Microtubule, *mTOR* Mechanistic target of rapamycin, *rPTH* Recombinant parathyroid hormone, *PD-1* Programmed death-1, *Rc* Receptor, *CDK* Cyclin dependent kinase

**Table 2:** This table depicts the median age (min–max) and gender distribution for the MRONJ cases reported to the FAERS database for each of the twenty most commonly reported medications from 2010 to 2014 and from 2015 to 2021

**2010 to 2014**			**2015 to 2021**		
**Medication:**	**Median Age (Min–Max)**	**% Female**	**Medication:**	**Median Age (Min–Max)**	**% Female**
1. Zoledronic Acid	65 (26–113)	59.7	1. Denosumab	71 (18–104)	65.3
2. Alendronate	67 (30–102)	89.8	2. Zoledronic Acid	68 (23–98)	59.8
3. Denosumab	70 (24–113)	57.3	3. Alendronate	76 (44–97)	89.3
4. Pamidronate	65 (35–113)	75.7	4. Ibandronate	75 (42–100)	93.5
5. Ibandronate	65 (30–93)	91.1	5. Lenalidomide	70 (43–94)	44.7
6. Lenalidomide	64 (34–87)	39.0	6. Pamidronate	64 (39–89)	67.0
7. Risedronate	66 (40–85)	91.4	7. Bevacizumab	64 (39–80)	72.8
8. Sunitinib	61.5 (29–80)	21.4	8. Prednisolone	71 (22–91)	55.3
9. Bevacizumab	60 (21–74)	62.0	9. Risedronate	77 (47–95)	90.1
10. Prednisone	70 (34–89)	50	10. Dexamethasone	69 (46–90)	38.4
11. Dexamethasone	64 (34–84)	45.7	11. Everolimus	64.5 (29–82)	27.8
12. Docetaxel	63 (52–83)	25	12. Sunitinib	61 (33–85)	20.5
13. Letrozole	67 (46–84)	100	13. Palbociclib	65.5 (43–90)	97.3
14. Methotrexate	69 (52–84)	93.3	14. Docetaxel	68 (33–86)	25.9
15. Everolimus	63.5 (52–77)	33.3	15. Methotrexate	66 (34–88)	70.6
16. Paclitaxel	65 (56–73)	88.9	16. Prednisone	65 (35–91)	63.8
17. Imatinib	72 (62–76)	75	17. Pomalidomide	70 (48–83)	48.3
18. Sorafenib	65.5 (40–76)	25	18. Radium 223	73 (61–86)	0
19. Teriparatide	72 (42–82)	100	19. Nivolumab	67 (22–79)	13.0
20. Temsirolimus	61 (49–70)	33.3	20. Cabozantinib	61 (40–79)	27.3

**Table 3:** Two-by-two contingency table for disproportionality/signal analysis [6]

Number of Cases	Osteonecrosis of the Jaw (ONJ)	All other Adverse Events (AE)
Specific AE associated with medication	A	C
All other AEs associated with medication	B	D

Reporting Odds Ratio (ROR) calculation: = (AD) / (BC)

**Table 4:** This table depicts the reporting odds ratio (ROR) for the top 10 reported medications from 2010–2014

Medication	Reporting Odds Ratio (ROR)
1. Zoledronic Acid	263.1
2. Alendronate	69.0
3. Denosumab	37.2
4. Pamidronate	294.8
5. Ibandronate	34.9
6. Lenalidomide	2.7
7. Risedronate	48.6
8. Sunitinib	5.9
9. Bevacizumab	3.5
10. Prednisolone	5.8

**Table 5:** This table depicts the reporting odds ratio (ROR) for the top 10 reported medications from 2015–2020

Medication	Reporting Odds Ratio (ROR)
1. Denosumab	129.3
2. Zoledronic Acid	380.9
3. Alendronate	80.2
4. Ibandronate	45.0
5. Lenalidomide	1.8
6. Pamidronate	255.7
7. Bevacizumab	6.2
8. Prednisolone	5.3
9. Risedronate	52.3
10. Dexamethasone	3.9

## Discussion

We identified 19,668 cases of MRONJ in the FAERS database from January 2010 to April 2021. We further narrowed our pool of cases after incorporating the above inclusion criteria and removing duplicates, resulting in 3,132 cases for time period 1 and 5,776 cases for time period 2. This study is primarily being compared to a similar study done by Zhang et al. in 2016 where 17,119 ONJ cases were identified when reporting the relationship between medications and ONJ [4]. We will refer to that prior study as the comparison study for the remainder of the discussion. Our study removed duplicate cases and used a stricter inclusion criteria, thus improving our accuracy of the data that was collected so the strength of our conclusions would be increased.

Previously identified drug classes associated with ONJ included bisphosphonates, RANKL inhibitors, antiangiogenic agents, and m-TOR inhibitors. The underlying mechanisms by which these antiresorptive agents, antiangiogenic agents, and m-TOR inhibitors cause MRONJ are under review. Bisphosphonates have generally been used to treat hypercalcemia secondary to bone resorption, osteoporosis, and other metabolic bone diseases [7]. Additionally, by virtue of their antiresorptive properties both bisphosphonates and denosumab impair the bone remodeling process via inhibition of osteoclast activity as well as induction of cell apoptosis [4]. Moreover, formation of new blood vessels, mediated through vascular endothelial growth factor (VEGF) is considered an essential component of bone remodeling; therefore, disruption of this step may also predispose patients to MRONJ development [4]. Everolimus, for example, an m-TOR inhibitor involved in cell growth and metabolism, decreases VEGF levels and inhibits the growth and proliferation of tumor cells, endothelial cells, fibroblasts, and blood vessels [8].

As seen in the comparison study, the majority of the identified reported cases of MRONJ had received treatment with bisphosphonates and RANKL inhibitors (followed by antiangiogenic agents such as bevacizumab, sunitinib, or sorafenib) [4]. Interestingly, each of the listed bisphosphonates in the comparison study composed a smaller percentage of the total number of cases identified within our study during the same 2010–2014 time period using our methodology. For example, zoledronic acid composed up to 67.1% of total number of cases in the prior study whereas our study indicated 60.8%; a similar downtrend was noted with alendronate from 42.7% to 18.4%. Denosumab, on the other hand, had an increasing proportion of the total number of cases, previously noted at 6.9% now at 16.2%. It is worth noting that the total number of ONJ cases affiliated with these medications were far less. The demographics described in the comparison study were similar, except for a slightly higher percentage of males and an older age noted in the current study (average age of 62.2 ± 12.1 years versus 66.1 ± 11.1 years respectively. Nine novel drugs were discovered in time period 1, which included the following: lenalidomide, corticosteroids (prednisolone and dexamethasone), docetaxel and paclitaxel, letrozole, methotrexate, imatinib, and teriparatide. These drugs were not previously mentioned in the comparison study.

The novel drugs that potentially are associated with MRONJ identified in this current study suggest additional mechanisms of actions contributing to the development of MRONJ. Chemotherapy has been implicated in previous case reports of MRONJ, but a clear association remains difficult to demonstrate as the majority of patients with underlying bone disorders are simultaneously receiving chemotherapy and bisphosphonates [9]. It can be theorized that bisphosphonate-related ONJ may be worsened by concomitant antineoplastic therapy. Lenalidomide, an immunomodulating agent with anti-neoplastic properties, has been shown to modulate the subtract specificity of CRL4CRBN E3 ubiquitin ligase via ubiquitination of transcription factors, IKZF1 and IKZF3; subsequent proteasomal degradation of these transcription factors kills multiple myeloma cells [10]. It has been studied that the ubiquitination-proteasome and degradation system is an essential process that has been involved in the regulation of cell proliferation, differentiation and survival, and dysregulations in this system lead to pathologies including cancers [11]. E3 ubiquitin ligases, the most abundant group of enzymes involved in the ubiquitination pathway, have been implicated in the regulation of bone cells through the degradation of receptor tyrosine kinases; disruption of this step via the use of lenalidomide may be theorized to predispose MRONJ development [11]. Other notable drug classes include corticosteroids, which can be used as a part of adjunctive therapy for malignancy and are commonly prescribed in cancer patients for a variety of symptoms. Glucocorticoids have been theorized to cause osteocyte apoptosis with resultant disruption of bone vascularity and diminution of bone hydraulic support [12]. The taxanes, including docetaxel and paclitaxel, are microtubule-targeting medications that help inhibit cancer cell proliferation by binding to microtubules and suppressing microtubule dynamics and leading to mitotic arrest; docetaxel has generally been indicated for metastatic prostate, breast, and other solid tumors whereas paclitaxel has been used for breast, ovarian, and non-small cell lung cancer [9]. In fact, Aragon-Ching et al. described a cohort of patients with metastatic prostate cancer who were treated with a multiagent regimen including docetaxel and two anti-angiogenesis agents. This group developed MRONJ with an incidence that far exceeded previous reports in the current literature [13]. Methotrexate has been known to modulate cell-specific signaling pathways involved with inflammation via promotion of adenosine release and inhibition of transmethylation reactions. It has been commonly used to treat certain types of malignancies and autoimmune disorders, such as rheumatoid arthritis [14]. We were able to identify another unique tyrosine kinase receptor inhibitor, imatinib, which has known anti-angiogenic properties and is most frequently used in the treatment of leukemia. Interestingly, teriparatide has actually been shown to improve the rate of resolution of MRONJ despite being listed as an associated drug with ONJ in the FAERS database. Teriparatide is a human recombinant peptide, which has been shown to enhance bone regeneration in elderly patients and noted to see improved bone markers in ONJ patients via its anabolic effects [15]. We hypothesize that teriparatide was most likely used for the treatment of MRONJ in these cases reported to the FAERS database and is unlikely to be a causative agent in the development of MRONJ.

While MRONJ remains a rare, but serious ADR, it has been suggested that incidence of MRONJ cases has risen with the development of new molecular target and immunological drugs in the treatment of malignancies [16]. In addition to these comparisons seen during time period 1 to the prior study, several drugs and drug classes that have not been previously described in literature are identified using FAERS data during time period 2. Drugs with known anti-neoplastic properties include: palbociclib, radium-223, nivolumab, pomalidomide, and cabozantinib. Other notable cancer medications with continued MRONJ association include: bevacizumab, sunitinib, everolimus, temsirolimus, and sorafenib. These drugs have been perceived to play a large role in suppression of bone turnover, angiogenesis inhibition, infection and inflammation, soft tissue toxicity, and immune-related [16].

Denosumab was found to be the most commonly reported medication associated with ONJ within the FAERS database for time period 2. Due to the descriptive nature of the study, incidence rates of ADRs cannot be calculated using the FAERS database [17]. When comparing the most commonly reported drugs from each time period there were significant similarities with multiple medications (except in modified order) with exception of sunitinib (listed as #8 in earlier time period transitioned down to #12 in the latter time period), as seen in Fig. 2. This overlap of commonly reported medications associated with ONJ for both time periods gives further evidence that these drugs are actually associated to the development of ONJ.

The medication that was most frequently reported for time period 1 was zoledronic acid, comprising approximately 60.8% of the cases of MRONJ. Of interest, denosumab was the highest reported drug for time period 2 (54.5% of cases). This change may be due to changes in drug prescription preference, as studies comparing denosumab to zoledronic acid have shown that denosumab is superior to zoledronic acid for delaying or preventing skeletal related events [18]. It is anticipated that females contribute to the majority of cases of MRONJ (as further illustrated in see Fig. 3), as females represent a target population for the treatment of osteoporosis. According to Alswat, women are more likely to receive osteoporosis preventative treatment (72% vs. 45%) and bisphosphonate was used in their treatment more frequently than compared to men (61% vs. 39%) in 2005 [19]. Thus, it is reasonable to infer that the majority of patients who experience MRONJ while on bisphosphonate therapy would be women stemming from the higher rates of use in females. Similar to the comparison study, the calculated total percentages of these medications is greater than 100% likely suggesting that some of these patients may have been on multiple drugs for ongoing treatment or patients may have changed from one drug to another. Interestingly, the percentages for the top three medications have changed significantly when analyzing the drug utilization patterns between the two different time periods. Although there was also a significant change in the number of cases over time, each of the time frames had a different number of cases to start with. For example, the percentage of Denosumab from time period 1 to time period 2 dramatically increased, whereas reports for Zoledronic acid and Alendronate percentages decreased. This trend perhaps suggests that ONJ is being recognized as a more common and serious side effect that can be avoided, and it is likely that we are seeing changes in its use as it is being recognized more now. In fact, a majority of the ROR for the drugs listed during time period 1 have shown to increase, likely indicating increased reporting within the FAERS database over time. This could be explained by increased use, surveillance, or number of cases. Although incidence cannot be inferred, it is a good indication that this is more commonly reported or caught in the setting of these medications. Unfortunately, the inability to remove duplicates from the overarching database limits the strength of the information that can be obtained with regards to the ROR.

There have been several novel medications that have been identified to be associated with ONJ during time period 2. Palbociclib, a cyclin-dependent kinase 4/6 inhibitor, has been previously studied to treat breast cancer. CDK 4/6 are downstream of multiple signaling pathways which lead to cellular proliferation. There have been 6 cases of MRONJ associated with these inhibitors, warranting awareness and further close monitoring for MRONJ with this drug class. [20]. Nivolumab, targets PD-1 receptor expressed on activated T cells and promotes antitumor immunity. Nivolumab has been effective for treating non-small cell lung cancer, melanoma, renal cell carcinoma and other cancers [21]. Pomalidomide, a thalidomide analogue with antineoplastic, anti-angiogenic and immunomodulatory properties has a role, in conjunction with dexamethasone in treatment of multiple myeloma and has been hypothesized as another potential association with MRONJ [22]. Sunitinib and imatinib are newer tyrosine kinase inhibitors with known antiangiogenic activity. Cabozantinib, another tyrosine kinase inhibitor with anti-angiogenic properties, was observed to be associated with ONJ when used in a 51-year-old female with medullary thyroid cancer [23]. Lastly, though not a common form of treatment for osteoporosis, studies have shown that radium-223 in conjunction with use of bisphosphonates/antiresorptive medications may have a synergistic role in the development of MRONJ [24]. Radium itself targets the body by incorporating into newly formed bone matrix within osteoblastic metastatic lesions via high-energy alpha particle and induces DNA double-strand breaks leading to cell death in nearby exposed tumor cells, osteoblasts, and osteoclasts [25].

Limitations of this study include the use or exposure to multiple medications, making it difficult to determine the individual causes of MRONJ as well as the clinical indication for these drugs. It would be difficult to elaborate on this detail given the fact that at times, many patients have used multiple drugs simultaneously during different time periods and it would be difficult to elicit which indication was associated with which drug. Particularly investigating one isolated drug would not reflect the data represented in our paper. Reports of medication-induced ONJ often include multiple medications. Our method to control for this was to list all medications in the list as potential contributors to ONJ. Regardless of including multiple medications, medications such Zoledronic were included in a majority of cases (60.8% during time period 1) compared to Pamidronate contributing to 9.6%. We believe that it is unlikely that separating out by individual would change the numbers only and perhaps represent a different but not necessarily better method of analyzing the data. However, it would be worthwhile and interesting to note this in future studies, and to modify our inclusion criteria for patients only receiving one drug. Another limitation of the study is the descriptive nature of information obtained from FAERS. By definition, FAERS is not used to measure the incidence of a specific ADR. Nonetheless, FAERS is specifically used to detect signals and describe the occurrences of reported ADRs within the known limitations of the database. Future studies using data from individual electronic medical records may be used to confirm the findings of our study and provide more data on the incidences of ONJ resulting from individual medications. Utilizing the Naranjo scoring algorithm can help evaluate for causality, however the large sample size of our study limits the ability to conduct individual chart review necessary to establish causality in all cases. A feasible approach to establishing causality in future studies could be to focus on smaller sample size or individual medications associated with ONJ. An additional limitation is that the reporting of ADRs is voluntary, with significant problems of the FAERS system including under-reporting and the potential for reporting bias. We sought to limit reporting bias by only reviewing reports to those from healthcare professionals.

## Conclusions

In conclusion, this updated analysis of the FAERS system for MRONJ has further clarified the medications associated with this ADR. In addition, the study has identified several medications that have not been previously described in the literature that may cause or have a contributing effect to MRONJ. In addition to the recognized association with bisphosphonates and RANKL-inhibitor (ie, denosumab), drug classes including other antineoplastic agents were found. Evaluation of the mechanism of action of these medications, including anti-angiogenesis, immunomodulation, cytotoxicity and hormonal effects may shed light on their potential to cause this devastating ADR. It would be interesting to investigate the specific anatomical origin of MRONJ for future studies. This can potentially be accomplished by specifying the anatomical region as part of the initial search filter. In this paper, we limited the search to “osteonecrosis of the jaw,” which was assumed to be associated with maxilla and/or mandible involvement, as the FAERS database cannot be used to differentiate the specific anatomic location of ONJ. Most importantly, a number of cases were identified that listed as mortality as the outcome within the database. Because patients with ONJ often have multiple comorbidities, these findings require further investigation to determine the actual role of ONJ in the reported mortalities.

## Data Availability

The dataset supporting the conclusions of this article is available via the public FDA Adverse Event Reporting System database found at https://www.fda.gov/drugs/questions-and-answers-fdas-adverse-event-reporting-system-faers/fda-adverse-event-reporting-system-faers-public-dashboard. The ‘FAERS Public Dashboard’ option was then selected prompting to its home page. The ‘Search’ tab was then selected, and the rest of the information can be found within the Methods section of the manuscript.

## Abbreviations

ONJ: Osteonecrosis of the jaw
ADR: Adverse Drug Reaction
FDA: Food and Drug Administration
FAERS: FDA Adverse Event Reporting System
MRONJ: Medication-related osteonecrosis of the jaw
AAOMS: American Association of Oral and Maxillofacial Surgeons
ROR: Reporting Odds Ratio
RANKL: Receptor activator of nuclear factor-kappa-B-ligand
RANKL-i: Receptor activator of nuclear factor-kappa-B-ligand inhibitor
BP: Bisphosphonate
MOA: Mechanism of action
TKI: Tyrosine kinase inhibitor
CS: Corticosteroid
MT: Microtubule
mTOR: Mechanistic target of rapamycin
rPTH: Recombinant parathyroid hormone
PD-1: Programmed death-1
Rc: Receptor
CDK: Cyclin dependent kinase
VEGF: Vascular endothelial growth factor

## References

[1] Ganesan K, Bansal P, Goyal A, et al. “Bisphosphonate.” StatPearls. Treasure Island (FL): StatPearls Publishing; updated 2021 Jul 6. Accessed date 26 Sept 2021.

[2] Saad F, Brown JE, Van Poznak C (2012). Incidence, risk factors, and outcomes of osteonecrosis of the jaw: integrated analysis from three blinded active-controlled phase III trials in cancer patients with bone metastases. Ann Oncol.

[3] American Association of Oral and Maxillofacial Surgeons. 2014. Medication-Related Osteonecrosis of the Jaw-2014 Update. Retrieved from https://www.aaoms.org/docs/govt_affairs/advocacy_white_papers/mronj_position_paper.pdf. Accessed date 10/07/2021.

[4] Zhang X, Hamadeh I, Song S (2016). Osteonecrosis of the Jaw in the United States Food and Drug Administration’s Adverse Event Reporting System (FAERS). J Bone Miner Res.

[5] Banda J, Evans L, Vanguri R (2016). A curated and standardized adverse drug event resource to accelerate drug safety research. Sci Data.

[6] Choi JY, Choi JH, Kim MG (2021). Signal Detection of Adverse Drug Reactions of Cephalosporins Using Data from a National Pharmacovigilance Database. Pharmaceuticals (Basel).

[7] Gupta, Mohit and Gupta, Neha. “Bisphosphonate Related Jaw Osteonecrosis.” StatPearls. StatPearls Publishing; updated 2021 Jul 28. Accessed date 7 Oct 2021. 30521192

[8] Yamamoto D (2017). Osteonecrois of the jaw associated with everolimus: A case report. Mol Clin Oncol..

[9] Azarenko O, Smiyun G, Mah J, et al. “Antiproliferative Mechanism of Action of the Novel Taxane Cabazitaxel as Compared with the Parent Compound Docetaxel in MCF7 Breast Cancer Cells.” Mol Cancer Ther. 2014;13(8):2092–2103. 10.1158/1535-7163.MCT-14-0265 24980947

[10] Fink EC, Benjamin LE (2015). The novel mechanism of lenalidomide activity. Blood..

[11] Sévère N (2013). E3 ubiquitin ligase-mediated regulation of bone formation and tumorigenesis. Cell Death Dis.

[12] Weinstein RS (2012). Glucocorticoid-induced osteonecrosis. Endocrine.

[13] Aragon-Ching BJ, Ning Y, Chen CC (2009). Higher incidence of osteonecrosis of the jaw (ONJ) in patients with metastatic castration resistant prostate cancer treated with anti-angiogenic agents. Cancer Invest.

[14] Komatani T, Sonobe J, Takahashi K (2017). Methotrexate-related osteonecrosis of the jaw: Report of two cases. J Oral Maxillofacial Surg Med Pathol.

[15] Kwon Y, Kim D (2016). “ Role of Teriparatide in Medication-Related Osteonecrosis of the Jaws (MRONJ). Dentistry J (Basel).

[16] NifosiFabrizio A, Zuccarello M, Nifosi L (2019). Osteonecrosis of the jaw in the era of targeted therapy and immunotherapy in oncology. J Korean Assoc Oral Maxillofac Surg.

[17] “FDA Adverse Event Reporting System (FAERS) Public Dashboard,” https://www.fda.gov/drugs/questions-and-answers-fdas-adverse-event-reporting-system-faers/fda-adverse-event-reporting-system-faers-public-dashboard.

[18] Stopeck AT, Lipton A, Body J-J (2010). Denosumab compared with zoledronic acid for the treatment of bone metastases in patients with advanced breast cancer: a randomized, double-blind study. J Clin Oncol.

[19] Alswat KA (2017). Gender Disparities in Osteoporosis. J Clin Med Res.

[20] Marcianò A, Guzzo GM, Peditto M (2020). Medication-Related Osteonecrosis of the Jaws and CDK4/6 Inhibitors: A Recent Association. Int J Environ Res Public Health.

[21] Guo L, Zhang H, Chen B (2017). Nivolumab as Programmed Death-1 (PD-1) Inhibitor for Targeted Immunotherapy in Tumor. J Cancer.

[22] Ayoade F, Olayiwola A, Li A (2018). Holes in the Jaw—A Report of Two Cases of Periapical Actinomycosis. Diseases.

[23] Marino R, Orlandi F, Arecco F (2015). Osteonecrosis of the jaw in a patient receiving cabozantinib. Aust Dent J.

[24] Cao Y, Trieu J, Rojas V, et al. “Osteonecrosis of the jaw (ONJ) in radium 223 (Ra223)-treated metastatic castration-resistant prostate cancer (mCRPC) patients (pts) with exposure to zoledronic acid and/or denosumab. J Clin Oncol. 2020;38(15):5575.

[25] Morris MJ, Corey E, Guise TA (2019). Radium-223 mechanism of action: implications for use in treatment combinations. Nat Rev Urol.

